# Humans Continue to Outperform Large Language Models in Complex Clinical Decision-Making: A Study with Medical Calculators

**Published:** 2024-11-08

**Authors:** Nicholas Wan, Qiao Jin, Joey Chan, Guangzhi Xiong, Serina Applebaum, Aidan Gilson, Reid McMurry, R. Andrew Taylor, Aidong Zhang, Qingyu Chen, Zhiyong Lu

**Affiliations:** 1 Division of Intramural Research, National Library of Medicine (NLM), National Institutes of Health (NIH), Bethesda, MD, USA.; 2 Department of Computer Science, University of Virginia, Charlottesville, VA, USA.; 3 Department of Ophthalmology & Visual Science, Yale School of Medicine, New Haven, CT, USA.; 4 Massachusetts Eye and Ear, Harvard Medical School, Boston, MA, USA.; 5 Department of Emergency Medicine, Boston Medical Center, Boston, MA, USA.; 6 Department of Biomedical Informatics & Data Science, Yale School of Medicine, New Haven, CT, USA.; 7 Department of Emergency Medicine, Yale School of Medicine, New Haven, CT, USA.

## Abstract

Although large language models (LLMs) have been assessed for general medical knowledge using medical licensing exams, their ability to effectively support clinical decision-making tasks, such as selecting and using medical calculators, remains uncertain. Here, we evaluate the capability of both medical trainees and LLMs to recommend medical calculators in response to various multiple-choice clinical scenarios such as risk stratification, prognosis, and disease diagnosis. We assessed eight LLMs, including open-source, proprietary, and domain-specific models, with 1,009 question-answer pairs across 35 clinical calculators and measured human performance on a subset of 100 questions. While the highest-performing LLM, GPT-4o, provided an answer accuracy of 74.3% (CI: 71.5–76.9%), human annotators, on average, outperformed LLMs with an accuracy of 79.5% (CI: 73.5–85.0%). With error analysis showing that the highest-performing LLMs continue to make mistakes in comprehension (56.6%) and calculator knowledge (8.1%), our findings emphasize that humans continue to surpass LLMs on complex clinical tasks such as calculator recommendation.

Large language models (LLMs) such as GPT-4 and Med-PaLM have achieved expert-level performance on biomedical tasks like medical question-answering (QA)^1–5^. While these LLMs have been evaluated using medical licensing exam questions to quantify their general medical knowledge^6–8^, their capability for promoting real-world clinical decision support (CDS) and going beyond factual recall cannot be fully assessed by medical exams alone^9^. Simultaneously, clinical calculators, i.e. evidence-based computational tools for risk assessment, prognosis, and diagnosis, play an important role in real-world clinical care delivery^10,11^ and are becoming increasingly prevalent via web-based platforms like MDCalc^12,13^. With recent research also showing that LLM agents can engage in tool learning^14,15^, clinical calculators represent a set of tools that may be used to augment medical LLM agents and their capability to probabilistically reason^16^. However, the capability of LLMs to reason through complex clinical scenarios and appropriately select calculators for recommendation remains unknown.

To address this gap, we conducted the first comprehensive analysis of whether LLMs can recommend clinical calculators, with comparison to human performance, and curated MedQA-Calc, a first-of-its-kind dataset for evaluating the clinical calculator recommendation capabilities of LLMs. To construct MedQA-Calc, we identified 35 popular clinical calculators from MDCalc, excluding questionnaire calculators as questionnaires can rely on subjective inputs not found in patient reports. We then curated questions relating to these calculators using publicly available patient data from case reports in PubMed Central^17^; this data source, PMC-Patients, covers a broad range of clinical settings (emergency medicine, inpatient care, outpatient clinic, and surgery) and includes patient cases from both the United States and international populations. Leveraging the case reports, we identified instances of clinical calculator usage within patient notes, truncated notes to remove evidence of calculators at the point of use, and constructed multi-choice questions using the extracted calculators as ground-truth. We curated 1,009 question-answer instances, where each instance contained the truncated patient note, a generalized question for recommending calculators, answer options, and a ground-truth answer.

Figure 1 presents the overall study design. Using PMC-Patients as a data source for publicly available patient case reports from PubMed Central, we leveraged GPT-4o and few-shot learning to extract clinical calculators from patient cases (methodology and code detailed at https://github.com/ncbi-nlp/Calculator-Recommendation). We then truncated the extracted texts to remove evidence of calculator usage by implementing a fine-tuned model of GPT-4o. Next, we curated questions by randomly incorporating five answer choices from our list of calculators, excluding calculator options that were similar to the ground-truth calculator. For evaluation, we focused on three procedures: (1) *LLM performance*, where model accuracy in answering questions was assessed; (2) *Human performance*, where two medical trainees answered a subset of curated questions; and (3) *Error analysis*, where researchers and medical trainees reviewed the types of errors made by LLMs across the questions used to evaluate human performance.

The results of the evaluation are shown in Figure 2. Both LLM and human performance were evaluated in “closed-book” settings as neither evaluation included the use of external tools like literature search engines or web browsing. Eight LLMs were used for evaluation, and two medical trainees were used for human evaluation. In terms of LLM performance, GPT-4o provided the best nominal stand-alone performance; although, GPT-4o performance is not significantly different from Llama-3–70B or GPT-3.5-Turbo performance. Additionally, the low performance of PMC-LLaMa suggests that our curated dataset is affected by minimal data contamination^18^. With human performance, two medical trainees provided the same answer choices for 69 out of 100 questions. One medical trainee (90.0%, CI: 84.2%–95.9%) significantly outperformed GPT-4o (74%, CI: 65.4%–82.6%) on the subset of questions, and one medical trainee (69.0%, CI: 59.9%–78.1%) matched performance with GPT-4o. Thus, LLMs show the potential to provide calculator recommendations on par with humans; however, they have not reached human-level performance, even at the medical training level, for the clinical task of calculator selection. To further investigate errors made by LLMs, we divided LLM errors into four groups: comprehension (misunderstanding or hallucination), calculator knowledge (incorrect calculator usage), alternative choice (choosing a different calculator option that may be appropriate), and no explanation (LLMs lacking sufficient explanation). To assess human error, medical trainees reviewed their errors and reported agreement with ground-truth answers. Reasons for disagreement included limited patient note vignettes and reasonable alternative answers.

Figure 2c displays the confusion matrices of GPT-4o and medical trainees. Depending on the annotator comparison, GPT-4o correctly answered at least four out of the 100 questions that medical trainees incorrectly answered. Overall, only six out of 100 questions were answered incorrectly by both medical trainees and GPT-4o. We next evaluated the performance errors. We found that high-performing models like GPT-4o most commonly made comprehension and alternate solution errors (Fig. 2d). In contrast, high-performing models made proportionally fewer errors due to incorrect calculator knowledge.

A closer look at GPT-4o explanations during error analysis revealed that advanced models may struggle with making inferences with respect to time. For example, GPT-4o may automatically assume that a patient, who has already received a Sequential Organ Failure Assessment (SOFA) score, does not require an additional SOFA score. Moreover, GPT-4o may fail to recognize the utility of using a calculator score for diagnostic support. If a truncated patient note reveals a respiratory diagnosis, GPT-4o may report that a respiratory risk calculator is not needed as a diagnosis has already been made (LLM explanations and data are publicly available at https://github.com/ncbi-nlp/Calculator-Recommendation). Ultimately, large language models may require further integration with time-series data to provide applicable, real-world clinical decision support.

Our study design has several limitations. Firstly, though we have manually evaluated the relevance of each calculator for each instance, clinical calculator selection may not always be necessary given evidence-based practices. In addition, though we designed our study to exclude similar calculators during answer choice curation, multiple distinct medical calculators may be appropriate in clinical practice. For example, the process of truncating patient reports at the first appearance of a given medical calculator can remove mentions of other pertinent calculators within the downstream text. Moreover, the size and scope of the dataset is limited as it uses only 35 medical calculators; thus, we excluded other medical calculators that may have appeared within patient texts. Finally, the PMC-Patients dataset is composed of case reports which may deviate from clinical scenarios where clinical calculators are more commonly applied; such case reports also span multiple countries, where disease burden, documentation, and practice patterns may vary.

In future work, we will address these limitations by expanding the coverage of clinical calculators or medical decision-making scenarios and creating more granular patient vignettes, stratified across medical specialties. Additionally, we will consider curating open-ended clinical calculator questions to evaluate the clinical utility of LLMs in medicine. We will also measure the baseline quantitative reasoning of LLMs as done in recent work with MedCalc-Bench, a benchmarking study on the capability of LLMs to perform medical calculations^19^. Finally, we will further integrate our dataset with LLMs through tool learning, a key feature of language agents. With works like OpenMedCalc and AgentMD using medical calculators to augment LLMs^16,20^, our dataset of explanations and calculator evidence will better inform the use of language agents and medical calculators.

In summary, we present the curation and evaluation of question-answer instances relating to medical calculator usage, a complex clinical task. In addition to GPT-4o demonstrating nominally lower multi-choice accuracy as compared to the average performance of medical trainees, large language models continue to show errors in comprehension and calculator knowledge. Despite filtering similar calculators during question-answer curation, our research also identified error cases in which other calculator answers may be appropriate. This suggests that further curation and evaluations in open-answer settings are needed to test the capabilities of LLMs in medical decision-making.

## Methods

### Data Source

We manually reviewed MDCalc in June 2024 to identify 35 medical calculators from MDCalc’s internal “Popular” list of calculators, to collect calculator names, and to curate calculator codes (e.g., abbreviating “Glasgow Coma Scale” as “gcs”. While popular calculators involving only questionnaire data were excluded from the evaluation, we gathered calculators across medical domains including cardiology, neurology, pulmonology, critical care, and surgery. Next, we extracted patient summary information from PMC-Patients, a publicly available dataset of real, anonymized case reports.

### Note extraction

Using GPT-4o, we extracted text evidence of calculators within each patient note, patient ID, calculator score values, calculator names, and calculator units. After data cleaning (i.e., limiting to 1000 instances per calculator, removing calculators not present in our list of 35 calculators), our dataset, MedQA-Calc, consisted of 7,768 medical calculator prediction instances.

### Note truncation

Then, we implemented a GPT-4o model (fine-tuned on 70 truncated patient-calculator note instances) to truncate patient notes at the appearance of any given extracted medical calculator name and value. An example of truncation is shown below:

#### Original note:

A 55-year-old African American male with a past medical history of hypertension presented to the emergency department at our hospital with a chief complaint of generalized weakness… His Wells score for pulmonary embolism (PE) was 7.5 putting him at high risk of PE.

#### Truncated note:

A 55-year-old African American male with a past medical history of hypertension presented to the emergency department at our hospital with a chief complaint of generalized weakness…

After running notes through the truncation model, we had a total of 7,758 medical calculator question-answer instances. We then split this data into a training and test set of 6,614 and 1,144 instances, respectively. We curated the testing set by including all calculator types, ensuring a maximum 50 instances per calculator.

### Question curation

Next, two researchers, NW and JC, manually reviewed the test instances to ensure that notes were properly truncated with respect to the original note and calculator evidence. After NW reviewed all instances, JC reviewed all validated instances.

After manual review, question-answer vignettes were randomly assigned answer options to include five options. Of note, we included “None of the above” as a possible answer choice in all cases, and we also created an exclusion matrix so that calculators with similar purposes (e.g., CURB-65 and Pneumonia Severity Index) were not provided alongside one another.

### Question evaluation

We evaluated eight LLMs (i.e., GPT-4o, GPT-3.5 Turbo, Llama-3–70B, Llama-3–8B, Mixtral-8×7B, Mistral-7B, Meditron, and PMC-Llama) using our dataset of 1,009 questions. Additionally, we employed two medical trainees to review 100 instances of calculator recommendations across all 35 calculators. Medical trainees answered questions in a closed-book setting with no external resources. After receiving ground-truth answers, medical trainees then evaluated calculator instances by labeling their agreement with the question-answer vignette.

### Error Analysis

In order to perform additional analysis of LLM errors, we had two annotators review the errors made by LLMs on the subset of 100 questions that they answered. Annotators labeled LLM errors as (1) comprehension errors, (2) calculator knowledge errors, (3) alternate solution errors, or (4) “no explanation” errors according to our provided annotation guidelines. Of note, “no explanation” errors were defined as errors made by LLMs that did not provide an explanation or rationale for answers. In cases of error type disagreement, annotators discussed the LLM error until a consensus was reached.

## Figures and Tables

**Figure 1:**
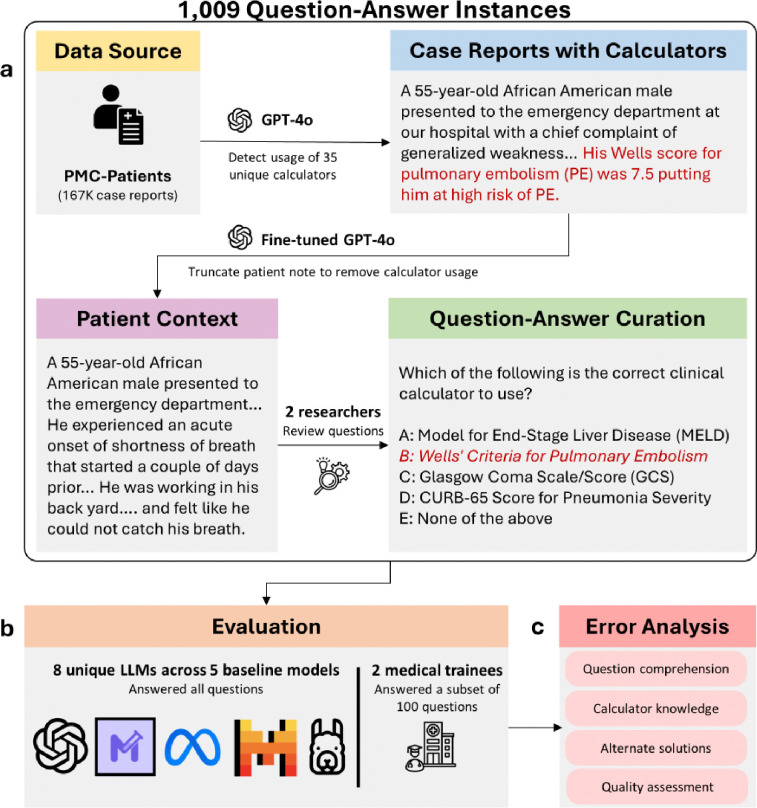
Study design. The curation and evaluation workflow for our medical calculator question-answering dataset, MedQA-Calc. **a.** Patient case reports containing calculators were identified with GPT-4o and transformed into questions using a fine-tuned GPT-4o model. Answer options were curated using automatic randomization, excluding similar answer choices. **b.** The capability of LLMs and medical trainees to answer these questions and recommend calculators was evaluated. **c.** Researchers and medical trainees reviewed LLM errors and evaluated the quality of questions.

**Figure 2.**
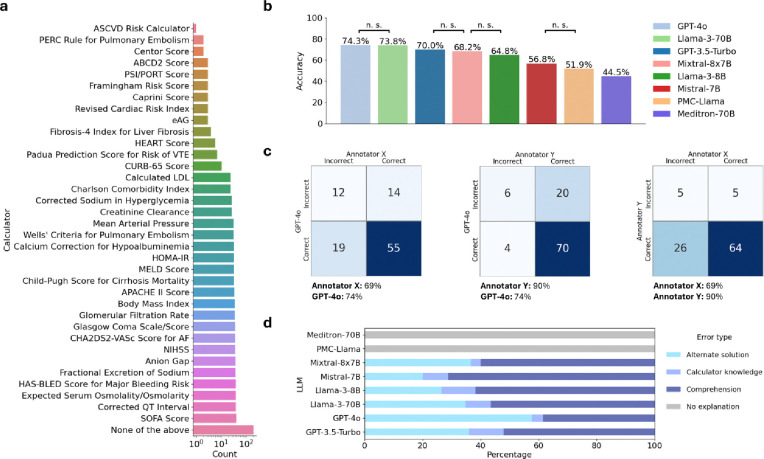
Evaluation results. The results of our curation and evaluation. **a.** The frequency of clinical calculators found in the MedQA-Calc dataset. **b.** The accuracy of different LLMs in answering our curated questions; n.s. not significant, all other accuracy differences are significant with p < 0.05. **c.** Confusion matrices representing the performance of two medical trainees and the highest-performing LLM on a subset of 100 questions. **d.** Bar graphs representing the proportions of errors made by each LLM model on a subset of 100 questions, stratified by error type (i.e., comprehension, calculator knowledge, alternative solution, no explanation).

**Table 1.** Extracted medical calculators, their abbreviations in Figure 2, and their codes used with LLMs.

Calculator Name	Calculator Abbreviation (Code)
ABCD2 Score	ABCD2 Score (abcd2)
The Acute Physiology and Chronic Health Evaluation II (APACHE II) score	APACHE II score (apache)
Anion Gap	Anion Gap (anion)
Atherosclerotic Cardiovascular Disease (ASCVD) Risk Calculator	ASCVD Risk Calculator (ascvd)
Body Mass Index (BMI)	Body Mass Index (bmi)
Calcium Correction for Hypoalbuminemia	Calcium Correction for Hypoalbuminemia (corrected_calcium)
Caprini Score for Venous Thromboembolism	Caprini Score (caprini)
Calculated LDL	Calculated LDL (ldl)
Centor Score (Modified/McIsaac) for Strep Pharyngitis	Centor Score (centor)
Charlson Comorbidity Index (CCI)	Charlson Comorbidity Index (cci)
CHA2DS2-VASc Score for AF	CHA2DS2-VASc Score for AF (cha2ds2)
Child-Pugh Score for Cirrhosis Mortality	Child-Pugh Score for Cirrhosis Mortality (child_pugh_score)
Corrected QT Interval	Corrected QT Interval (qtc)
Corrected Sodium in Hyperglycemia	Corrected Sodium in Hyperglycemia (corrected_sodium)
Creatinine Clearance (Cockcroft-Gault Equation)	Creatinine Clearance (creatinine)
CURB-65 Score for Pneumonia Severity	CURB-65 Score (curb65)
Expected Serum Osmolality/Osmolarity	Expected Serum Osmolality/Osmolarity (osmo)
Fibrosis-4 (FIB-4) Index for Liver Fibrosis	Fibrosis-4 Index for Liver Fibrosis (fib4)
Fractional Excretion of Sodium (FENa)	Fractional Excretion of Sodium (fena)
Framingham Risk Score	Framingham Risk Score (framingham)
Glasgow Coma Scale/Score (GCS)	Glasgow Coma Scale/Score (gcs)
Glomerular Filtration Rate (GFR)	Glomerular Filtration Rate (gfr)
HAS-BLED Score for Major Bleeding Risk	HAS-BLED Score for Major Bleeding Risk (has_bled)
HEART Score	HEART Score (heart)
HOMA-IR (Homeostatic Model Assessment for Insulin Resistance)	HOMA-IR (homa_ir)
HbA1c to Estimated Average Blood Glucose Conversion (eAG)	eAG (eag)
Mean Arterial Pressure (MAP)	Mean Arterial Pressure (map)
Model for End-Stage Liver Disease (MELD) Score	MELD Score (meld)
National Institutes of Health Stroke Scale/Score (NIHSS)	NIHSS (nihss)
Padua Prediction Score for Risk of VTE	Padua Prediction Score for Risk of VTE (padua)
PERC Rule for Pulmonary Embolism	PERC Rule for Pulmonary Embolism (perc)
PSI/PORT Score: Pneumonia Severity Index for CAP	PSI/PORT Score (psi_port)
Revised Cardiac Risk Index for Pre-Operative Risk	Revised Cardiac Risk Index (card_risk)
Sequential Organ Failure Assessment (SOFA) Score	SOFA Score (sofa)
Wells’ Criteria for Pulmonary Embolism	Wells’ Criteria for Pulmonary Embolism (wells_pe)

## Data Availability

The PMC-Patients dataset is publicly available through Hugging Face datasets at https://huggingface.co/datasets/zhengyun21/PMC-Patients. The MedQA-Calc dataset is available at https://huggingface.co/datasets/Nicholas-Wan/MedQA-Calc.
